# Effects of Plant Substitutes for Nitrite on the Technological Characteristics of Fermented Sausages: A Comprehensive Review

**DOI:** 10.1002/fsn3.70186

**Published:** 2025-04-18

**Authors:** Sima Tahmouzi, Behnam Alizadeh Salmani, Soheyl Eskandari, Masoumeh Arab

**Affiliations:** ^1^ Student Research Committee, School of Public Health Shahid Sadoughi University of Medical Sciences Yazd Iran; ^2^ Department of Food Sciences and Technology, School of Public Health Shahid Sadoughi University of Medical Sciences Yazd Iran; ^3^ Food Science Department, School of Nutritional Sciences and Dietetics Tehran University of Medical Sciences (TUMS) Tehran Iran; ^4^ Food and Drug Laboratory Research Center (FDLRC), Food and Drug Administration (IR‐FDA) Ministry of Health and Medical Education (MOH + ME) Tehran Iran; ^5^ Department of Food Hygiene and Safety, Zoonotic Diseases Research Center, School of Public Health Shahid Sadoughi University of Medical Sciences Yazd Iran; ^6^ Research Center for Food Hygiene and Safety, School of Public Health Shahid Sadoughi University of Medical Sciences Yazd Iran

**Keywords:** fermented sausages, meat industry, nitrates and nitrites, plant‐based alternatives, technological characteristics

## Abstract

Meat and its derivatives stand out as valuable sources of premium proteins, essential B‐complex vitamins, and minerals. The processing of raw meat, a common practice for creating diverse products like sausages and hams, traditionally involves the use of curing salts, predominantly sodium nitrates and nitrites These salts confer several advantages, encompassing color stabilization, inhibition of spoilage‐causing microorganisms such as *Clostridium perfringens* , *Clostridium botulinum*, and enhancement of the final product's flavor and aroma. Despite their benefits, the utilization of curing salts raises concerns about potential health risks, particularly the association with an increased risk of esophageal, stomach, and bladder cancers due to the formation of nitrosamine hormone‐like chemicals. To mitigate the intake of nitrites and nitrates, various natural alternatives, including spinach, celery, radish, lettuce, carrots, and beets, have been proposed. This review critically evaluates plant‐based substitutes for nitrates and nitrites, examining their influence on the quality, flavor, microbial communities, and physicochemical properties of fermented sausages. By delving into these alternatives, the review aims to contribute valuable insights into developing healthier and more sustainable approaches in the processing of fermented sausages.

## Graphical abstract

Since the consumption of sodium nitrite is harmful to humans and this substance is used as an additive in fermented sausages, it is suggested to use plant substitutes for nitrite. In this article, it deals with a comprehensive review of the proposed plant substitutes and the different effects of these substitutes on the technological characteristics of fermented sausages.

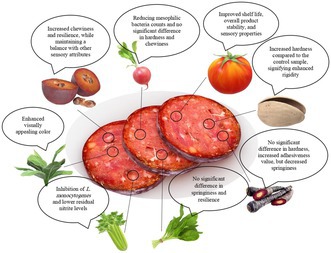

## 1 Introduction

The history of fermented sausage production dates back thousands of years. The origin can be traced to Southern Europe, specifically the Mediterranean region, and some Asian countries (Flores and Piornos 2021). These sausages undergo curing through the fermentation of a meat mixture, followed by drying and curing facilitated by beneficial bacteria. This transformative process not only prevents the proliferation of harmful bacteria but also imparts a distinctive flavor to the sausages (Bou et al. 2017). Producing fermented sausages entails utilizing small and large cuts of raw meat combined with fat, water, and additives such as salts (including nitrates and nitrites), flavorings and seasonings, and fillers (Franciosa et al. 2018). Subsequent to fermentation, the sausage is encased and left to hang, initiating the drying and curing phase. Depending on the desired flavor and texture, this stage can extend for several weeks to months (Toldrá 2017). Across various cultures, fermented sausages have gained popularity, with each region contributing unique styles and flavors. Renowned examples include Salami, Chorizo, and Pepperoni (Flores and Piornos 2021; Hwang et al. 2023; Vignolo et al. 2010). The classification of fermented sausages encompasses two primary groups: dry sausages, characterized by a water activity (*a*_w_) less than 0.9, and semidry sausages, with an aw ranging from 0.9 to 0.95. Additionally, based on pH levels, these sausages can be categorized as either low acid (final product with pH of 6) or high acid (final product with pH of 3.5 or less). This nuanced approach to classification reflects the diversity and complexity inherent in the world of fermented sausage production (Comi et al. 2005; Vignolo et al. 2010).

Nitrite holds a pivotal role in meat products, exerting substantial influence on their sensory attributes and overall organoleptic characteristics while serving as a potent agent in deterring oxidation and microbial proliferation, either in isolation or when synergistically combined with other additives (Škrlep et al. 2022). Numerous studies have revealed nitrite's dual role as an antioxidant (oxidative compounds like nitroso‐ and nitrosyl compounds) and its importance for meat quality (Karwowska et al. 2019; Shakil et al. 2022). The antioxidant properties of nitrite are believed to be connected to the creation of cured color, which involves interactions with heme proteins and metal ions, as well as the ability of nitric oxide to counteract free radicals. The interaction of nitrite with myoglobin instigates the formation of nitrosyl myoglobin, resulting in a bright red color in the meat (Parthasarathy and Bryan 2012). In terms of microbial safety, nitrite acts as a formidable inhibitor against pathogenic agents like *Clostridium botulinum* , disrupting crucial iron–sulfur enzymes, including ferredoxin and hydrogenase. Consequently, nitrite hinders the production of acetyl phosphate by influencing the phosphor‐trans acetylate enzyme (Alahakoon et al. 2015; Ford and Lorkovic 2002). However, it is imperative to acknowledge that excessive nitrite consumption poses adverse health effects, elevating the risk of gastrointestinal, stomach, and intestinal cancers, as well as blood pressure concerns attributable to nitrosamine production (Milkowski et al. 2010). Organic meat products in the EU are limited to a maximum of 80 ppm of nitrites, according to Regulation EC 780/2006. In the United States, however, the use of nitrites and nitrates in organic products is prohibited entirely (Lopez et al. 2021). Current Serbian legislation limits the maximum allowable concentrations of nitrate and nitrite in processed meat, expressed as sodium nitrite (NaNO_2_) or sodium nitrate (NaNO_3_), to 100 and 150 mg/kg, respectively, depending on the specific type of product (Milešević et al. 2022). In France, the maximum permitted levels are 120 ppm for sodium nitrite combined with 120 ppm for sodium nitrate, or 200 ppm for sodium nitrate in products that have been dried for more than 3 weeks (Bonifacie et al. 2021). In China, the maximum dosage of sodium nitrite and nitrate in fermented meat is 30 ppm, and in India, the permissible limit for sodium nitrite is 80 ppm. In Mexico, the permissible limit for sodium nitrate and nitrite is 156 ppm, while in Argentina it is 150 ppm (Zhang et al. 2023).

This underscores the delicate balance between reaping the benefits of nitrite and safeguarding human health (Ferysiuk and Wójciak 2020; Karwowska and Kononiuk 2020).

Recently, the meat industry has been actively exploring alternatives to nitrates and nitrites, driven by a quest for more sustainable and health‐conscious options. Achieving this objective involves employing diverse technologies, including high‐pressure and high‐heat frequency processes (Zhang et al. 2023). Additionally, the incorporation of antibacterials like bacteriocin and the utilization of plant‐based nitrate sources represent promising avenues in this endeavor (Ferysiuk and Wójciak 2020; Toldrá 2006). Plant‐based alternatives of nitrite, derived from sources like celery, radish, lettuce, carrots, and spinach, offer a natural and clean‐label solution in the meat industry (Alahakoon et al. 2015; Zhang et al. 2023). These vegetables contain naturally occurring nitrates, which can be converted into nitrites during processing. The benefits of utilizing these plant‐based alternatives include enhanced safety, as they eliminate concerns associated with synthetic nitrites and a potential reduction in the risk of certain health issues. Moreover, incorporating these sources aligns with consumer preferences for more sustainable and plant‐derived ingredients, contributing to the development of healthier and environmentally conscious meat products (Shakil et al. 2022). In order to no study was conducted on reviewing the effects of plant extracts on safety and quality of fermented sausages, the study aims to provide an in‐depth understanding of how plant‐derived (extrac, powder and essentional oil) may influence the microbial, textural, color, and sensory properties of fermented sausages.

## 2 Nitrates and Nitrites: Advantages and Disadvantages

Nitrate (${\mathrm{NO}}_{3}^{-}$) and nitrite (${\mathrm{NO}}_{2}^{-}$) are naturally occurring compounds found in a variety of foods, especially in vegetables and fruits (Ferysiuk and Wójciak 2020; Zhang et al. 2023). They are also commonly used as food additives in the form of sodium nitrate (NaNO_3_) and sodium nitrite (NaNO_3_) which are essential for the preservation and color enhancement of cured meats such as bacon, ham, and sausages (Alahakoon et al. 2015; Pini et al. 2020). The widespread use of nitrates and nitrites in meat products is attributed to their numerous advantages, particularly their antimicrobial properties (Liu et al. 2017).

Nitrates and nitrites are effective agents in inhibiting the growth of harmful pathogens, such as *C. botulinum* , which is responsible for botulism, a severe and potentially fatal foodborne illness (Jo et al. 2020). Through the inhibition of bacterial growth, nitrates and nitrites extend the shelf life of meat products, ensuring prolonged safety for consumption. Moreover, these compounds play a pivotal role in shaping the color and flavor profile of cured meats like bacon and ham. During the curing process, the introduction of these compounds leads to the formation of nitric oxide and nitric acid, which interact with myoglobin in the meat to produce a stable pinkish‐red pigment known as nitrosylhemochrome (Govari and Pexara 2015; Shakil et al. 2022).

The pink color developed in cured meats not only enhances the product's visual appeal but also serves as a crucial indicator for consumers, signaling proper curing and safety. A study conducted by Sindelar and Milkowski (2012) examined the effects of varying levels of nitrates and nitrites on color development in cured meats. Their findings revealed that the incorporation of these compounds resulted in the formation of the desirable pink hue, with higher concentrations of nitrates correlating with a more intense color saturation (Sindelar and Milkowski 2012).

In the production of fermented sausages, nitrates fulfill a distinct and vital role. During the fermentation process, the nitrates present in the sausage are converted into nitrites through microbial activity, particularly by specific bacterial strains such as *Staphylococcus* and *Micrococcus* (Berardi et al. 2021). This conversion results in nitrites that are crucial for inhibiting the growth of spoilage microorganisms and pathogens, thereby ensuring the safety and integrity of the final products. Additionally, nitrite acts as a stabilizing agent for fatty acids, helping to prevent rancidity and off‐flavors. It also contributes to the formation of aromatic compounds that enhance the characteristic flavor profile of fermented sausages (Keeton 2017; Xu and Zhu 2021).

A study conducted by Ruiz‐Capillas and Jiménez‐Colmenero (2004) investigated the influence of nitrates and nitrites on the microbiological quality and sensory characteristics of fermented sausages. The findings indicated that the controlled addition of these compounds significantly enhanced sensory attributes, extended shelf life, and improved safety by effectively suppressing microbial growth, particularly that of Staphylococcus (Ruiz‐Capillas and Jiménez‐Colmenero 2004).

Despite the numerous benefits associated with the use of nitrates and nitrites, their application in the meat industry is subject to certain limitations (Tang et al. 2023). A significant concern is the potential formation of nitrosamines, which are recognized carcinogens, when these compounds are exposed to high cooking temperatures, such as during grilling or frying (Pereira et al. 2012). Additionally, nitrates are unstable in acidic environments, leading to their decomposition into nitrites and nitrogen dioxide. As a result, nitrites—derived from both nitrate metabolism and dietary sources—can react in the gastrointestinal tract with precursors of N‐nitroso compounds (NOCs), such as amines and amides, ultimately resulting in the formation of NOCs (Eisenbrand et al. 2022).

**FIGURE 1:**
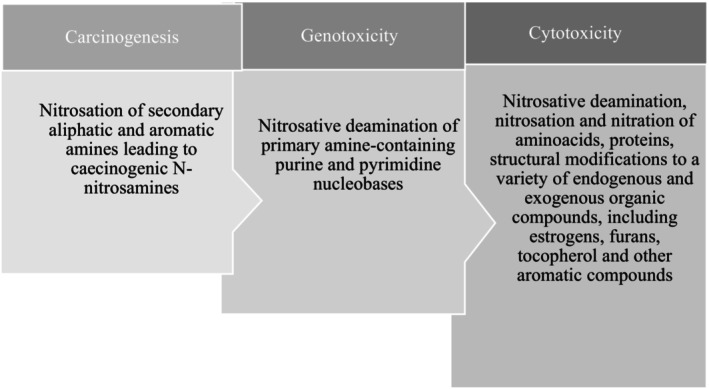
Toxicological effects of reactive nitrogen species.

The formation of N‐nitroso derivatives by nitrates and nitrites has been associated with higher incidences of cancer (such as lung cancer, breast cancer, and gastric cancer), the incidence of leukemia, brain tumors, and nasopharyngeal problems when they are present at high levels (Seyyedsalehi et al. 2023). Research indicates that patients with lung cancer exhibit significantly higher serum concentrations of nitrated proteins, which confirms the occurrence of nitrosative stress (Dalle‐Donne et al. 2006). Furthermore, proteins are susceptible to modification by reactive nitrogen species. When proteins are exposed to these species, substantial physical alterations occur in their structure, leading to various functional consequences. These include inhibition of binding and enzymatic activities, increased susceptibility to aggregation and proteolysis, and altered immunogenicity (Moldogazieva et al. 2018; Souza et al. 2001) (Figure 1).

The potential health hazards associated with nitrates and nitrites in processed meats have prompted regulatory authorities to implement stringent limits on the permissible levels of these compounds. A meta‐analysis conducted by Di et al. (2023) examined the relationship between processed meat consumption and the risk of colorectal cancer. The study found a positive correlation between high intake of processed meats containing nitrates and nitrites and an increased risk of colorectal cancer, attributing this link to the potential formation of nitrosamines during cooking (Di et al. 2023). Additionally, another concern arises from the increased sodium intake that can result from combining nitrates and nitrites with sodium chloride during curing processes. This is particularly problematic for individuals with hypertension or those at risk for cardiovascular diseases, highlighting the urgent need to explore alternative preservation methods in meat processing (Pegg and Shahidi 2008; Shahidi and Pegg 1992).

**FIGURE 2:**
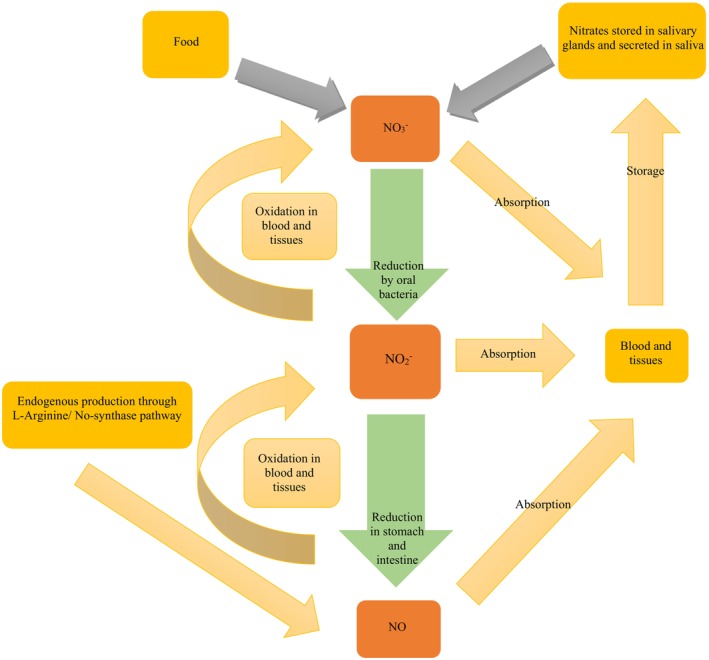
Simplified scheme of nitrite and nitric oxide (NO) metabolism in the body.

The formation of carcinogenic nitrosamines is a significant concern for scientists, manufacturers, and consumers alike. The harmful effects of nitrite as a meat additive were first documented in the early 1950s and 1960s with the identification of nitrosamine compounds (NOCs) (Bedale et al. 2016; Gassara et al. 2016). In the acidic environment of the stomach, nitrates are converted into nitric oxide (NO) and various metabolites. Subsequently, in the upper part of the small intestine, nitrates are absorbed into the bloodstream and eventually secreted by the salivary glands (refer to Figure 2) (Habermeyer et al. 2015; Tamme, Reinik, and Roasto 2010).

Saliva contains nitrate concentrations that are approximately 10 times higher than those found in blood, as the salivary glands actively excrete nitrate. Furthermore, oral bacteria, particularly those residing on the tongue—such as Veillonella species, *Staphylococcus epidermidis* , and Actinomyces species—are responsible for reducing about 20%–25% of nitrate to nitrite, which accounts for roughly 5% of the total nitrate ingested (Bondonno et al. 2015). However, Tamme, Reinik, and Roasto 2010 indicates that approximately 70%–80% of total nitrite exposure results from the bacterial reduction of nitrate, with ingested nitrate serving as the primary source for nitrite conversion (Tamme, Reinik, and Roasto 2010).

**FIGURE 3:**
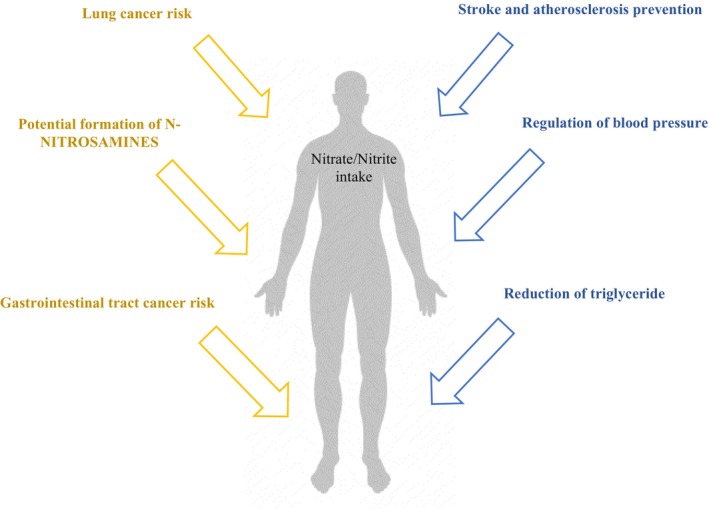
Benefits and adverse effects of nitrate/nitrite intake on the human body.

To address health concerns associated with nitrates and nitrites, government agencies such as the U.S. Food and Drug Administration (FDA) have established stringent regulations regarding their permissible levels in food products. The acceptable daily intake (ADI) for nitrate is set at 0–3.7 mg/kg body weight per day, which reflects the maximum allowable amounts of these compounds as food additives during processing (Karwowska et al. 2019). Currently, the permitted concentration of nitrite in processed meat is 150 mg/kg, while a lower limit of 100 mg/kg applies to sterilized meat products. Sodium nitrate is authorized solely for use in uncooked meats, with a maximum limit of 150 mg/kg. In the United States, the regulatory limit for nitrite in food products is established at 156 ppm, whereas Canada permits up to 200 ppm for similar products (Govari and Pexara 2015) (Figure 3).

## 3 Plant‐Based Alternatives

Nitrate assumes a vital role as a crucial nutrient within the realm of plants (Wang et al. 2018). Plant‐derived nitrate refers to the naturally occurring nitrate found in various plant‐based food sources, including leafy greens, beets, radishes, celery, and carrots. Plants absorb nitrate from the soil and undergo a transformative process that converts it into other nitrogen‐containing compounds necessary for their growth and metabolic functions (Dechorgnat et al. 2010; Liu et al. 2014).

The quantity of nitrate in plants depends on various factors, such as seed type, moisture content, storage duration, vegetable size, cultivation practices, growing season, and rainfall patterns (Anjana and Iqbal 2007). It is important to note that nitrate levels tend to decrease during storage and cooking processes (Ferysiuk and Wójciak 2020; Karwowska and Kononiuk 2020). Leafy vegetables, such as arugula, lettuce, and spinach, typically contain higher nitrate concentrations compared to seeds or tubers. However, beets and celery also exhibit significant levels of nitrate (Ferysiuk and Wójciak 2020).

Noteworthy investigations have systematically gauged the nitrate content across diverse vegetables, offering a comprehensive overview presented in Table 1.

**TABLE 1:** The nitrate content of different vegetables.

Sources	Total nitrate content (mg kg^−1^)	References
Spinach	723	Ximenes et al. (2000)
	2584	Tamme, Tamme, Reinik, Roasto, Meremäe, and Kiis (2010)
	2090	Tamme, Reinik, and Roasto (2010)
Radish	1297	Ximenes et al. (2000)
Celery	1036	Beheshti et al. (2023)
Broccoli	775	Ximenes et al. (2000)
	395	Hord et al. (2009)
	394	Nunez de Gonzalez et al. (2015)
Lettuce	1945	Ximenes et al. (2000)
	1303	Guadagnin et al. (2005)
	1074	Sušin et al. (2006)
Parsley	2813	Dejonckheere et al. (1994)
Beets	102.30–1619.80	Brzezińska‐Rojek et al. (2023)
Beetroots	1211	Ysart et al. (1999)
Carrot	282	Chung et al. (2003)
	264	Sušin et al. (2006)
Potato	158	Sušin et al. (2006)

Several research studies have examined the impact of substituting nitrate and nitrite salts with plant‐based alternatives in meat products. In addition to nitrates, vegetables contain a wide range of bioactive compounds, including phenolic compounds, organic acids, and flavonoids, which exhibit significant antioxidant and antimicrobial properties (Alahakoon et al. 2015). However, the influence of various factors on the results of using plant alternatives should not be ignored. The nature of the meat product itself, the intricacies of the production process, and the quantity of plant substitutes incorporated all emerge as pivotal determinants shaping the ultimate effects (Ferysiuk and Wójciak 2020).

Beetroot is recognized as a highly nutritious vegetable, offering a wide array of health benefits. It is rich in essential minerals and vitamins, contributing positively to health through its antioxidant properties, regulation of blood pressure, enhancement of digestive health, and potential anticancer effects (Chen et al. 2021; Thiruvengadam et al. 2022). Notably, beetroot boasts a nitrate concentration of 25 mg/100 g wet weight (Brzezińska‐Rojek et al. 2023). This concentration can vary based on several factors, including environmental conditions and cultivation practices (Gamba et al. 2021).

The potential of beetroot powder as a nitrate substitute in fermented beef sausage, specifically sucuk, was explored. The researchers incorporated varying concentrations of beetroot powder into different sausage formulations and evaluated both the physicochemical properties and sensory characteristics of the products. The findings demonstrated that the inclusion of beetroot powder significantly enhanced the color stability of the sausages, evidenced by a marked increase in redness (a*) values. Additionally, the nitrate content in the sausages was reduced, showcasing the potential of beetroot powder as an effective nitrate alternative. Sensory evaluations revealed no significant differences between the beetroot‐treated sausages and the control samples, suggesting consumer acceptability (Sucu and Turp 2018).

Radishes present a promising alternative to sodium nitrite in the production of fermented sausages, primarily due to their substantial nitrite content (Raczuk et al. 2014; Uddin et al. 2021). Furthermore, radishes are rich in phenolic compounds and vitamin C, which have been shown to exhibit antioxidant properties (Goyeneche et al. 2015). Their nutritional profile is further enhanced by a significant fiber content, along with essential vitamins and minerals such as calcium and potassium (Gamba et al. 2021). Additionally, radishes contain beneficial compounds like glucosinolates and isothiocyanates, which may aid in regulating blood sugar levels and enhancing liver function (Jaafar et al. 2020).

In this regard, Ozaki, Dos Santos, et al. (2021) and Ozaki, Munekata, et al. (2021) investigated the use of radishes and beets as alternatives to nitrite in fermented sausages, focusing on their physicochemical and microbial characteristics. The findings indicated that treatments incorporating beet powder resulted in an increase in redness (a* values), while no significant differences were observed in total nitrate retention across the treatments. Additionally, the levels of lactic acid increased during storage. Sensory evaluations revealed no differences between the treated samples and the control group over a period of 84 days (Ozaki, Munekata, et al. 2021). Similarly, Jin et al. (2018) reported that the inclusion of radish powder and oregano essential oil (OEO) enhanced both the physicochemical properties and sensory stability of the sausages compared to untreated samples (Jin et al. 2018).

Tomato has been investigated as a potential alternative to nitrite in the formulation of fermented sausages, providing not only distinctive flavor contributions but also beneficial properties. Although specific data on the nitrate content of tomato powder is lacking, studies indicate that fresh tomatoes typically contain nitrate levels ranging from 0.93 to 66.54 mg/kg, with an average concentration of 12.55 mg/kg (MirMohammad‐Makki and Ziarati 2015).

Tomatoes are rich in antioxidants, particularly lycopene, which imparts the characteristic red color. Lycopene is known for its ability to delay oxidation due to its quenching properties (Collins et al. 2022; Frusciante et al. 2007). In a study conducted by Skwarek and Karwowska (2022), tomato pomace was used at a 0.2 mg/kg concentration as an alternative to nitrite in fermented sausages. The antioxidants in tomatoes play a crucial role in protecting the lipids from oxidative degradation, contributing to improved shelf life and overall product stability (Skwarek and Karwowska 2022).

Eyiler and Oztan (2011) investigated the use of tomato powder as a nitrite substitute in frankfurter formulations. The results indicated that the pH of the frankfurters treated with tomato powder was significantly reduced, and the oxidation process in these samples was effectively delayed. Furthermore, the incorporation of tomato powder enhanced the sensory properties of the frankfurters, suggesting its potential as a natural alternative to nitrite (Eyiler and Oztan 2011).

Celery, recognized for its high fiber and mineral content, has found application as a substitute for nitrate salts in the meat industry. Its antioxidant compounds, flavonoids, and vitamin C contribute to retarding oxidation in meat products (Horsch et al. 2014; Sowbhagya 2014). Organic meat producers in the US are permitted to use processed celery powder as a natural source of nitrates/nitrites within restricted quantities (0.2%–0.4% of the overall product weight) (Cerveny et al. 2009). In higher concentrations, unpleasant flavors are more likely to occur (Alahakoon et al. 2015).

Parsley, renowned for its historical medicinal and culinary uses, emerges as a viable nitrite replacement in fermented sausages. With essential oils (EOs), phenylpropane, terpene compounds, flavonoids, polyphenolic compounds, furanocoumarins, carotenoids, and various vitamins and minerals, parsley adds flavor and health benefits (Ajmera et al. 2019; Duthie 1999). The nitrate content in parsley can exceed 1500 mg/kg (Tamme, Reinik, Roasto, Meremäe, and Kiis 2010). In a study by Rail et al. (2017), the incorporation of parsley into fermented sausages resulted in sensory and textural properties comparable to those of control samples (Riel et al. 2017).

Black carrots, characterized by acylated anthocyanins, phenolic compounds, and vitamin C, offer stability and nutraceutical advantages (Kamiloglu et al. 2018). Anthocyanins are the significant bioactive compounds in black carrots, associated with their health‐promoting and disease‐preventing properties (Akhtar et al. 2017). carrots also contain nitrates at 200–250 mg/kg (Bender et al. 2020). Investigated the physicochemical, sensory, and microbial changes of fermented sausages containing black carrot concentrates (BCCs). The findings indicated that BCCs had a notable effect on sensory characteristics and color (a*). The samples with black carrots were deemed satisfactory regarding sensory properties (Ekici et al. 2015).

Various researchers have explored the potential of plant extracts and EOs as substitutes for nitrate and nitrite salts in meat products (Sardarodiyan and Mohamadi Sani 2016). EOs are volatile natural compounds commonly used in culinary applications to enhance flavor and impart health benefits to foods. Additionally, they serve as preservatives due to their diverse array of bioactive compounds, which exhibit antibacterial, antifungal, and antioxidant properties. Furthermore, EOs are generally recognized as safe (GRAS) (Burt 2004; Calo et al. 2015; Chivandi et al. 2016).

Several studies have assessed the effects of EOs in fermented sausages, both individually and in conjunction with plant‐based substitutes. While vegetables serve as a natural source of nitrates, challenges remain regarding their effectiveness in fulfilling the functions typically associated with nitrites. Consequently, the use of EOs has gained popularity due to their diverse bioactive compounds, which can effectively replicate the functions of nitrites (Al‐Maqtari et al. 2022).

Ozaki, Dos Santos, et al. (2021) and Ozaki, Munekata, et al. (2021) showcased the effectiveness of radish powder and OEO as sodium nitrite substitutes in cooked fermented sausages, yielding favorable sensory outcomes (Ozaki, Dos Santos, et al. 2021).

The combination of sodium nitrite (75 mg/kg) with the essential oil of *Juniperus communis* L. (0.01–0.10 μL/g) significantly enhanced the physicochemical, antioxidant, and antibacterial properties of fermented sausages (Tomović et al. 2020). Similarly, the incorporation of coriander essential oil (0.12 μL/g) alongside sodium nitrite (60 mg/kg) resulted in improved sensory attributes, particularly in terms of color, as well as enhanced oxidative quality and microbial stability (Šojić et al. 2019).

Caraway ( *Carum carvi* L.) essential oil (0.01, 0.05, 0.10 μL/g) was also used in a mixture with sodium nitrite (0, 75, 150 mg/kg) in dry‐fermented sausages. Results showed the oxidative stability of caraway essential oil, but the taste of the final product was not acceptable (Tomović et al. 2022). de Oliveira et al. (2012) investigated the effects of winter savory essential oil ( *Satureja montana* L.) on mortadella‐type sausages in different amounts of sodium nitrate and evaluated the color and antioxidant properties of the final products. Results showed that using EOs in concentrations less than 1.56% with sodium nitrate (100 mg/kg or lower) produces mortadella‐type sausages with the required qualities, which can meet the demands of consumers (de Oliveira et al. 2012).

## 4 Effects of Plant‐Based Alternatives on Microbial Characteristics of Fermented Sausages

One of the primary challenges facing the meat industry is the widespread issue of cross‐contamination and microbial spoilage caused by pathogenic bacteria and fungi, particularly yeast and mold. This concern is particularly pronounced in the production of fermented sausages, where it arises during multiple stages of meat processing (Flores and Piornos 2021; Tomović et al. 2020).

Nitrite, a commonly employed curing agent, plays a pivotal role in mitigating these microbial challenges. Its antimicrobial efficacy is intricately linked to the inhibition of bacterial metabolic enzymes, restriction of oxygen uptake, and disruption of the proton gradient. Notably, nitrite has demonstrated effectiveness in controlling the growth of *C. botulinum* (Alahakoon et al. 2015) and has been documented in multiple studies for its impact on the proliferation of *Listeria monocytogenes* (Riel et al. 2017).

**FIGURE 4:**
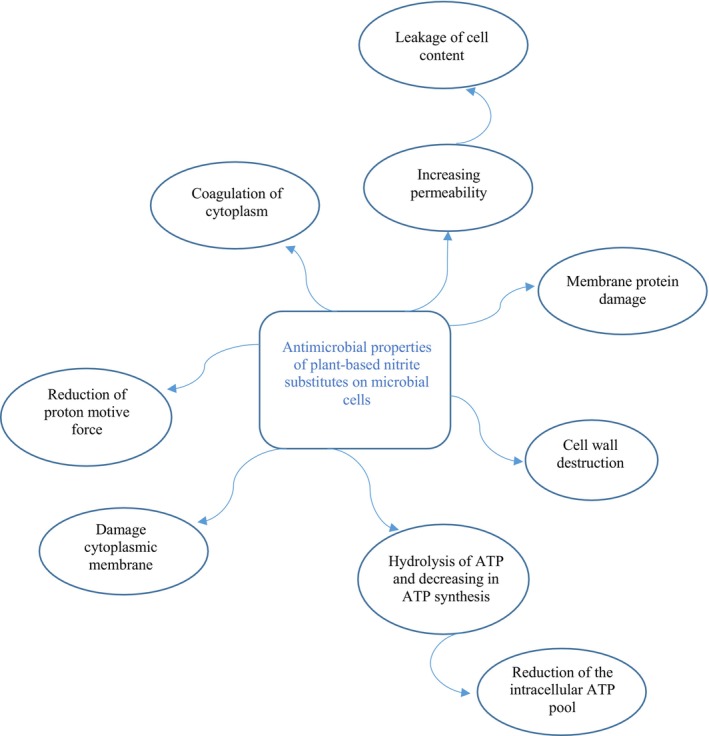
Antimicrobial properties of plant‐based substitutes on microbial cells.

Since the meat industry seeks to transition away from chemical additives in favor of plant‐derived alternatives, it becomes imperative for these substitutes to fulfill the requisite criteria, especially in terms of microbial and oxidative protection. A crucial aspect of this shift involves assessing the antimicrobial efficacy of plant‐based alternatives on microbial cells, as illustrated in Figure 4 (Hamdi et al. 2018; Karwowska and Kononiuk 2020).

Tahmouzi et al. (2012) delved into the impact of beet sugar (B.S.), calcium lactate (CL), and *Staphylococcus xylosus* on uncured frankfurters. After 2 months, samples with elevated B.S. and *S. xylosus* (8 logs viable cfu/g) exhibited minimal residual nitrite. Notably, higher B.S. and CL concentrations correlated with the absence of *Clostridium perfringens* during storage, showcasing their preservative potential. The nitrate‐reducing activity of B.S. and the effectiveness of CL were highlighted, suggesting their utility as preservatives in uncured frankfurters. The incorporation of *S. xylosus* , CL, and B.S. agents was deemed conducive to mitigating microbial risks and chemically enhancing frankfurters, encompassing improvements in flavor, texture, and shelf life (Tahmouzi et al. 2012).

Riel et al. (2017) investigated the effects of parsley extract powder on the microbial and sensory characteristics of Mortadella‐type sausages. The study found that the inhibition of *L. monocytogenes* and microbial spoilage in sausages cured with parsley extract powder was comparable to that observed in conventionally cured sausages containing more than 60 ppm nitrate. Furthermore, the use of parsley extract powder resulted in lower residual nitrite levels, presenting a potential strategy for reducing nitrite intake among consumers. While these findings are promising, further research into the antimicrobial activity of parsley extract against a broader range of microorganisms is essential for a more comprehensive understanding (Riel et al. 2017).

As mentioned in the previous section, EOs could meet the need for alternative additives to maintain the safety and quality of meat products (Ahmad et al. 2022). In another study, Ozaki et al. assessed radish powder and OEO in nitrite‐free fermented cooked sausages over 30 and 60 days of storage at 4°C and 20°C. The findings revealed an absence of *Salmonella* spp. in all samples, with coagulase‐positive *Staphylococcus* and sulfite‐reducing *clostridia* levels consistently below 10^2^ and 10 CFU/g, respectively. The results of total coliforms were below 3 MPN/g after 8 days of processing and during storage. It was effective in reducing mesophilic bacteria counts (2.3–2.4 log CFU/g in samples with 0.5% radish powder) and exhibited commendable sensory attributes (Ozaki, Dos Santos, et al. 2021).

In a related study, Meira et al. (2017) explored the efficacy of essential oil compounds and phenolic acids, including allyl isothiocyanate, carvacrol, ferulic acid, o‐coumaric acid, and p‐hydroxybenzoic acid, in dry‐fermented sausage production. Sausage batches contaminated with *Escherichia coli* O157:H7 underwent weekly physicochemical, microbial, and sensory analyses. Investigations showed that all phenolic acids tested synergized with allyl isothiocyanate against *E. coli* O157:H7, whereas only ferulic acid was synergistic with carvacrol. In advance, the combination of allyl isothiocyanate and ο‐coumaric acid at 20 × FIC (Fractional Inhibitory Concentration index) displayed a substantial reduction of *E. coli* O157:H7, complying with North American legislation. This combination is proposed as a viable alternative in the production of fermented sausages (Meira et al. 2017).

Grape seed extract (GSE), chestnut extract (CHE), and hydroxytyrosol (extracted from defatted olive pomace) have shown potential for antioxidant activity and microbial inhibition (Dave and Ghaly 2011; Farnaud and Evans 2003). Aquilani et al. (2018) manufactured Dry‐fermented pork sausages with two mixtures of natural antioxidants consisting of (i) GSE and olive pomace hydroxytyrosol and (ii) CHE and olive pomace hydroxytyrosol and evaluated the microbial, physicochemical, and sensory properties. They found that the major foodborne pathogens ( *E. coli* , *L. monocytogenes* , *Staphylococcus* spp., *Clostridium* spp., and *Salmonella* spp.) were absent or below the limit required in all samples, which indicated that these two natural substances would likely replace sodium nitrite in dry‐fermented sausages (Aquilani et al. 2018).

## 5 Effects of Plant‐Based Alternatives on Textural Attributes of Fermented Sausages

Fermented sausages constitute a unique category of cured meat products characterized by a complex and controlled fermentation process. The textural qualities of these sausages, which are highly valued by consumers, are influenced by various factors, including fermentation duration, moisture and fat content, protein composition, the types and quantities of additives used, and the conditions during the drying phase (Hwang et al. 2023; Ikonić et al. 2016).

During the fermentation process, starter microorganisms play a crucial role by lowering the product's pH through the production of lactic acid (Roca and Incze 1990; Van Ba et al. 2016). This acidification leads to the denaturation of protein structures, resulting in the condensation of muscle fiber proteins and the formation of a gel matrix. This gel structure significantly enhances the hardness and elasticity characteristics observed in fermented sausages (Hwang et al. 2023; Ikonić et al. 2016).

Furthermore, research has demonstrated that the fat content of fermented sausages significantly influences their texture, particularly regarding hardness and chewiness. Specifically, higher fat content has been associated with a decrease in both hardness and chewiness, thereby adding complexity to the intricate interplay of components that determine the overall texture of these products (Cruxen et al. 2018).

Table 2 illustrates the impact of herbal alternatives on the texture of fermented sausages. Beetroot powder ( *Beta vulgaris* ), known for its high nitrate content, was utilized by Sucu and Turp (2018) as a texture stabilizer in their study. The reformulation of dry sucuk involved substituting nitrite with beetroot powder, which was added at varying concentrations (0.12%, 0.24%, and 0.35%) alongside sodium nitrite at levels of 150, 100, and 50 mg/kg in the meat blend. The analysis focused on physicochemical attributes, pH levels, and textural profiles, employing texture profile analysis (TPA) using a two‐fold compression method with a texture analyzer. The results indicated that the addition of these relatively high concentrations of additives did not significantly affect the textural characteristics of the products (Sucu and Turp 2018). This investigation provides valuable insights into the potential of beetroot powder as a substitute for sodium nitrite, shedding light on its limited impact on the textural properties of fermented sausages.

**TABLE 2:** Textural attributes of plant‐based nitrite substitutes in fermented sausages.

Nitrite substitute	Sample	Levels	Results	References
Beetroot powder	Turkish fermented beef sausage (sucuk)	0.12% beetroot powder +100 mg/kg sodium nitrite, 0.24% beetroot powder +50 mg/kg sodium nitrite, and 0.35% beetroot powder	Hardness, springiness, cohesiveness, gumminess, chewiness, and resilience properties did not significantly change at the beginning and the end of the storage period	Sucu and Turp (2018)
Pistachio hull extract (PHE)	Fermented sausage	500, 700, and 1000 ppm	The 500‐ppm treatment had no significant difference in texture parameters from the control treatment The 750‐ppm treatment had less chewiness than the control sample The 100‐ppm treatment had more hardness than the control sample	Lashgari et al. (2020)
Parsley extract powder	Mortadella‐type sausages	1.07, 2.14, and 4.29 g/kg	There was no difference in hardness, resiliency, and chewing between treatments	Riel et al. (2017)
Celery powder	Chorizo	0.25%, 0.5%, 1%, and 2%	In the samples without NO, a reduction of hardness was detected	Martínez‐Zamora et al. (2021)
*Juniperus communis* L. essential oil	Dry‐fermented sausages	0.01, 0.05, 0.1 μL/g	There was no adverse effect on hardness, springiness, cohesiveness, and chewiness	Tomović et al. (2020)
Black carrot	Turkish dry‐fermented sausage	0.5, 1, 2 g/100 g	No significant difference in hardness, but decreased adhesiveness, springiness, cohesiveness, gumminess, chewiness, and resilience values were reported	Ekici et al. (2015)
Combination of radish powder and oregano essential oil (OEO)	Fermented cooked sausages	Radish powder: 0.5% and 1.0% OEO: 100 mg/kg	No significant difference in hardness and chewiness between treatments and control was observed. However, springiness and cohesiveness decreased compared to the control sample containing 150 mg/kg of sodium nitrite	Ozaki, Dos Santos, et al. (2021)
Lentil isolates	Fermented sausages	1%, 3%, and 7%	Results indicated that at a concentration of 3% lentil‐based protein isolate, sausages exhibited improved textural properties, including enhanced firmness and reduced crumbliness, without compromising sensory acceptance	Benković et al. (2023)
Combination of chitosan and radish powder	Fermented cooked sausages	Radish powder: 0.5% Chitosan: 0.25%–0.5%	Hardness, springiness, cohesiveness, and chewiness were lower than the control sample containing 150 ppm sodium nitrite	Ozaki et al. (2020)
Pea protein isolate	Traditional fermented sausages	1%, 2%, and 5%	Results indicated that a concentration of 2% pea protein isolate led to improved firmness and cohesiveness compared to the control samples	Molfetta et al. (2022)
Fermented soy sauce	Traditional fermented chorizo	1%–3%	The researchers observed improved cohesiveness and reduced hardness with the addition of 2% soybean powder, striking a favorable textural balance	Choi et al. (2019)
Combination of grape seed extract (GSE) and chestnut extract (CHE)	Dry‐fermented Italian Cinta Senese sausages	1%, 2%, 3%, and 4%	The researchers observed that an optimal concentration of 4% GSE and 2% CHE imparted desirable textural improvements, such as increased chewiness and resilience, while maintaining a balance with other sensory attributes	Pini et al. (2020)
Spinach extract	Italian dry‐fermented sausage	0.1%, 0.2%, and 0.3%	Italian sausages enriched with 0.3% spinach extract displayed textural attributes, including springiness and resilience, similar to those achieved with nitrite	Pennisi et al. (2020)
Combination of olive leaf extract (OLE) and nitrate/nitrite (NO)	Fermented sausages	CTRL (0 mg/kg OLE; 300 mg/kg NO), Tr1 (200 mg/kg OLE; 150 mg/kg NO), Tr2 (400 mg/kg OLE; 150 mg/kg NO), Tr3 (800 mg/kg OLE; 150 mg/kg NO), Tr4 (200 mg/kg OLE; 0 mg/kg NO), Tr5 (400 mg/kg OLE; 0 mg/kg NO), and Tr6 (800 mg/kg OLE; 0 mg/kg NO)	At the end of the ripening period, all the samples were within hygienic limits and the substitution of the additives with OLE allowed the reduction of NO residual contents In absence of NO, a significant reduction of weight loss was observed. Moreover, in the samples without NO a reduction of the hardness was detected The stability test showed that the increase of the OLE amount prolonged the induction time	Difonzo et al. (2022)
Lettuce	Fermented sausages	5%, 7%, 10%, and 15%	Results indicated that a 10% inclusion of the lettuce‐based sample led to a desirable texture profile, maintaining the characteristic firmness associated with traditional nitrite	Alahakoon et al. (2015)
Tomato pomace extract	Raw fermented sausages	0.2 mg/kg	Results indicated that formulations with 0.2 mg/kg tomato pomace extract exhibited enhanced textural firmness compared to the control group employing conventional nitrite The presence of polyphenolic compounds in tomato pomace was attributed to these improvements in texture	Skwarek and Karwowska (2022)
Arugula and barberry extract	Heat‐treated fermented sausage	—^a^	At the end of the storage, no differences were observed on the textural characteristics and the overall acceptability of all samples	Serdaroğlu et al. (2023)

^a^ Not mentioned.

Riel et al. (2017) showcased the positive impact of incorporating parsley extract powder as an alternative ingredient in mortadella‐type sausages, particularly influencing their texture profile. Initial TPA analysis (1‐day post‐production) revealed no significant differences in hardness, resiliency, and chewiness among the sausage variants (Riel et al. 2017).

In another study, pistachio hull was investigated as a substitute for sodium nitrite. Pistachio hull is recognized as a rich source of natural antioxidants and antimicrobial compounds, making it a cost‐effective bioactive substance that can play a significant role in the processing of fermented sausages (Hadidi et al. 2022). Lashgari et al. (2020) identified some characteristics of pistachio hull extract (PHE) with three levels of 500, 750, and 1000 ppm in fermented sausages. Beyond antimicrobial and antioxidative benefits, the study delved into the textural profile, encompassing hardness, cohesion, chewing ability, elasticity, and gummy state. Results demonstrated increased hardness compared to the control sample, signifying enhanced rigidity. Notably, the treatment containing 750 ppm PHE stood out in texture analysis, surpassing the other samples (Lashgari et al. 2020). This underscores the potential of PHE as a valuable component in optimizing the textural qualities of fermented sausages.

Black or purple carrots have emerged as a natural source of food colorants, prompting research into their potential impact on various properties of sucuk. Ekici et al. (2015) investigated the effect of raw BCC on the microbiological, textural, and aroma properties of sucuk. Notably, textural measurements revealed subtle differences in specific textural characteristics (hardness, adhesiveness, cohesiveness, and chewiness), with a particular emphasis on the adhesiveness values of the samples (Ekici et al. 2015).

However, research by Harun et al. (2022) introduced a different perspective. Their study explored the addition of Carrot‐thyme and carrot‐rosemary Eos to dry‐fermented sausages during storage. Intriguingly, the findings indicated that such additions could lead to increased hardness in the texture of the sausages (Harun et al. 2022). These contrasting results underscore the complexity of interactions between raw BCC and sucuk properties. While Ekici et al. highlight nuanced textural variations, Harun et al. bring attention to the potential for texture hardening when incorporating specific EOs during storage.

Ozaki et al. (2020) investigated the effects of adding radish powder (at concentrations of 0.5% and 1.0%) and OEO at 100 mg/kg to fermented cooked sausages produced without nitrite. The study evaluated the hardness, springiness, cohesiveness, and chewiness of the resulting products. The findings indicated no significant differences in textural parameters among the various treatments (Ozaki et al. 2020). This research offers valuable insights into the complex interactions among ingredients, providing a potential pathway for enhancing the textural qualities of processed meat products.

## 6 Effects of Plant‐Based Alternatives on Color Attributes of Fermented Sausages

Meat color represents a crucial indicator of product freshness and quality, playing a pivotal role in consumer attraction (Mancini and Hunt 2005; Troy and Kerry 2010). The primary pigments responsible for meat color are myoglobin and hemoglobin. Myoglobin exhibits three distinct color states: myoglobin, oxymyoglobin, and methemoglobin, with iron as ferro ions in myoglobin and oxymyoglobin (Ferysiuk and Wójciak 2020). The desirable bright red color on fresh meat surfaces is attributed to oxymyoglobin formation, while a thin layer just beneath, termed “Metmyoglobin,” exhibits a bright red hue due to oxidation (Danijela et al. 2013; Tomasevic et al. 2021).

Ascorbic acid is a reducing agent used in the meat process to convert the nitrite compound into nitric acid, contributing to the desirable red color of raw meat (Alahakoon et al. 2015). The reaction between nitric acid and iron with myoglobin and methemoglobin causes the formation of a red color. Concurrently, nitrous oxide interacts with myoglobin during processing to produce nitrosyl myoglobin, an unstable bright red compound that acts as a pigment in processed meats (Gassara et al. 2016). A recommended 2–14 ppm sodium nitrite is deemed sufficient for processed meat color (Alahakoon et al. 2015).

However, various factors impact meat color changes, including ripening, leading to nitrosomyoglobin formation and increased a* index. Water content influences brightness, with higher levels correlating to an increased L* index. Microorganisms, particularly Staphylococcus species, play a role in color alteration through oxygen consumption and metabolite production, affecting the b* index. Understanding these intricacies contributes to the nuanced control of meat color in processed products (Martínez‐Zamora et al. 2021; Tomović et al. 2020).

Therefore, careful consideration of meat product color is essential when selecting a substitute for nitrite. Among the available plant‐based alternatives, leafy greens rich in nitrates present promising options for replacing traditionally added nitrates in meat products. Their incorporation could reduce or even eliminate the need for nitrite in fermented sausages (Ferysiuk and Wójciak 2020). Additionally, various compounds found in these substitutes can significantly influence the coloration of meat products. For instance, it has been reported that bioactive compounds in the essential oil of *J. communis* enhance the L* value by interacting with myoglobin (Tomović et al. 2020).

In a study by Pennisi et al. (2020), spinach powder, which is rich in nitrates, was utilized as a substitute for traditional nitrites in the production of fermented sausages. The results indicated a significant reduction in nitrite content while preserving the desirable red color of the sausages. The effective concentration of spinach extract was determined to be 2% by weight, demonstrating its potential to maintain color stability (Pennisi et al. 2020).

Similarly, research conducted by Hwang et al. (2018) explored the use of celery powder as a natural source of nitrates in fermented sausages. The study found that a concentration of 3% celery powder effectively replaced traditional nitrites while providing an attractive color to the sausages (Hwang et al. 2018).

These findings underscore the diverse options available from vegetable substitutes and emphasize the importance of fine‐tuning concentrations to achieve optimal color outcomes in fermented sausages. Table 3 presents further studies regarding the color of fermented sausages with vegetable substitutes.

**TABLE 3:** Effects of plant‐based nitrite substitutes on color parameters of fermented sausages.

Nitrite substitute	Sample	Storage time (day)	Levels	Results	References
Combination of radish and beetroot powder	Fermented dried sausages	0, 7, 15, 30, 45, and 60 days	Radish: 0.5% and 1% Beetroot: 0.5% and 1%	The L* index of radish powder‐based and beetroot powder‐based samples increased and decreased in the processing step and storage time, respectively compared to the control sample Samples with beetroot and radish powder, except for sample containing 1% radish powder, had a higher a* index than the control sample in the processing stage. All samples had a higher a* index during storage time than the control sample Unlike beetroot, the b* index of radish powder‐based samples was higher than that of the control sample	Ozaki, Munekata, et al. (2021)
Beetroot powder	Sucuk	0, 56, and 84 days	0.12%, 0.24%, and 0.35%	Samples containing 0.24% and 0.35% of beetroot powder had a higher L* index than the control sample on day 84 The a* index of all beetroot‐based treatments was higher than the control sample on different days The b* index was higher in samples with concentrations of 0.12% and 0.24% of beetroot powder compared to the control sample on day 84	Sucu and Turp (2018)
Black carrots powder	Sucuk	12	0.5, 1, and 2 g/100 g	Exterior surface color: The treatment with 0.5 g of black carrot alone and, together with 150 mg of sodium nitrite, had higher L*, a*, and b* indexes Cut surface color: Only the treatment with 0.5 g of black carrot in combination with 150 mg of sodium nitrite had a higher L* index than the control sample All treatments had a lower a* and b* indexes than the control sample	Ekici et al. (2015)
Catechin extract	Raw‐cured sausages	1, 10, 20, and 30	300 mL/kg	The treatment containing 125 mg/kg of sodium nitrite combined with 300 mg of catechin extract showed a higher L*, a*, and b* indexes	Moawad et al. (2012)
Combination of tomato pomace extract and organic peppermint essential oil	Cooked pork sausages	1, 20, 40, and 60	Tomato pomace extract: 0.150, 0/075 μL/g Organic peppermint essential oil: 0/075 μL/g	Treatment T1 (100 mg/kg sodium nitrite) and T3 (50 mg sodium nitrite +0.150 μL/g tomato pomace) had the lowest and highest L* index, respectively Treatment T2 (50 mg of sodium nitrite) had a higher amount of a* index, and treatment T4 (50 mg of sodium nitrite +0.075 μL/g of tomato pomace) had the highest amount of b* index	Šojić et al. (2020)
Combination of radish powder and chitosan	Fermented cooked sausages	0, 10, 15, 30, 45, and 60	0.5% radish powder + 0.25%, 0.5% chitosan	The a* and b* indexes of the samples containing chitosan and radish were lower than the control sample in the processing and storage stages, while the L* index of the samples containing chitosan and radish was higher than the control sample in all stages	Ozaki et al. (2020)
*Juniperus communis* L. essential oil	Dry‐Fermented Sausages	14 days	0, 0.01, 0.05 and 0.10 μL/g	L* and b* decreased with increasing concentration of JEO, while a* increased with increasing the concentration	Tomović et al. (2020)
Caraway ( *Carum carvi* L.) EO	Low‐fat fermented sausages	21 days	0.01, 0.05, 0.10 μL/g	Caraway (0.1 μL/g) and sodium nitrite (75 mg/kg) had a synergistic effect in increasing the redness of low‐fat fermented sausages	Tomović et al. (2022)
Sage ( *Salvia officinalis* L.) EO	Dry‐fermented sausages	225 days	0.00, 0.05, and 0.10 μL/g	The indices of L* and a* did not differ significantly between Sage‐ and sodium nitrite‐based samples, while with the increase in the Sage concentration, the b* value decreased	Šojić et al. (2021)
Bay leaf extract ( *Laurus nobilis* L.) extract	Sucuk	60 days	1, 5, and 10 mL/kg	The L* index of samples containing 1 mL/kg extract was equal to the samples containing 5 mL/kg of this extract. While samples containing 5 mL/kg extract showed a higher a* index and a lower b* index than other treatments	Benli et al. (2024)
Winter savory ( *Satureja montana* L.) EO	Mortadella‐type sausages	30 days	7.80, 15.60, and 31.25 μL/g	The addition of EO decreased L* and a* and increased b*	Šojić et al. (2023)

## 7 Effects of Plant‐Based Alternatives on Sensory Properties of Fermented Sausages

Enhancing the sensory attributes of meat products is as important as addressing microbial safety, textural properties, and other relevant factors (Gómez et al. 2020). As a result, in‐depth exploration of sensory evaluation has become a central focus in numerous research studies. Researchers recognize that the overall quality of a meat product is not solely defined by quantitative measures such as microbial stability or textural integrity; significant attention has been devoted to understanding and improving its sensory dimensions. Factors such as flavor, aroma, and overall palatability play a crucial role in consumer satisfaction (Legako et al. 2016).

This realization has prompted numerous scholarly investigations into meat products' distinct sensory properties. Research into sensory evaluation aims to uncover consumer preferences, thereby improving meat product formulations to better align with consumer preferences.

The incorporation of plant‐based nitrite alternatives into fermented sausages creates a complex interplay of factors that significantly affect their sensory properties, including flavor, aroma, and overall palatability. The selection of plant‐derived nitrite substitutes directly influences the flavor profile, introducing distinct taste nuances that may vary based on the specific alternative used. Similarly, aromas are affected, as unique olfactory notes can emerge from the interactions of plant‐based compounds during fermentation. These changes in flavor and aroma can enhance the overall palatability of the sausage, thereby shaping the consumer's sensory experience (Rodrigues et al. 2023). As the industry strives for cleaner labels and sustainable practices, understanding and optimizing the sensory impact of plant‐based nitrite alternatives in fermented sausages is pivotal in meeting consumer expectations for both health‐conscious and enjoyable meat products.

In the study conducted by Ekici et al. (2015), the sensory evaluation of sucuk samples infused with BCC was performed at room temperature (25°C). Eight trained panelists, comprising staff and graduate students from the Food Engineering Department at Erciyes University, assessed the samples based on color, flavor, aroma, texture, and overall acceptability. The fermented sucuk samples were cut into small pieces and grilled for 1 min on each side before being served to the panelists. Notably, all sucuk samples enriched with BCC received favorable sensory scores, demonstrating consistent acceptability across varying concentrations of BCC and BCC/nitrite combinations (Ekici et al. 2015).

Meira et al. (2017) performed a sensory evaluation of dry‐fermented sausages containing a combination of essential oil compounds and phenolic acids by 56 untrained volunteers and a nine‐point hedonic test from 1 (disliked extremely) to 9 (liked extremely) to assess the five attributes. The results of the sensory analysis revealed that both treatments achieved higher scores across the five categories compared to the control treatment. Notably, no parameters received negative scores when juxtaposed with either treatment in isolation, affirming the positive impact of the essential oil compounds and phenolic acids on sensory perception (Meira et al. 2017).

To evaluate the efficacy of PHE in fermented sausages, Lashgari et al. (2020) produced treated sausages and investigated sensory characteristics such as taste, odor, and overall acceptability and then compared them with the control samples. The results showed that PHE affected the hardness and chewiness of samples. Finally, the study results claimed that PHE could be a natural antioxidant and preservative that increases the quality of fermented sausages (Lashgari et al. 2020).

Moreover, the effect of a combination of radish powder and OEO on color, *a*_w_, weight loss, and sensory characteristics of fermented cooked sausages was investigated by Ozaki et al. (2020). The treated products exhibited enhanced safety features and improved sensory properties. Notably, the sensory analysis highlighted that, despite the absence of nitrite in the sausage formulation, the combination of radish powder and OEO garnered positive consumer acceptance for all treatments (Ozaki, Dos Santos, et al. 2021).

Similarly, sensory properties (color, aroma, flavor, texture, and overall acceptability) of fermented cooked sausages with radish powder and two levels of chitosan were evaluated by Ozaki et al. (2020) using a nine‐point structured hedonic scale. Regardless of the concentration of chitosan employed, noticeable alterations were observed in key parameters such as color, flavor, and overall acceptability. The impact on these sensory attributes was consistent across various concentrations of chitosan, highlighting the significant influence of this compound on the perceived characteristics of the product (Ma and Ledward 2004). These findings underscore the potential of natural alternatives in advancing both the safety and sensory aspects of fermented sausages.

Sensory assessments of sucuks infused with *Thymbra spicata* L. EO at concentrations of 300 and 500 ppm were conducted by a panel of 47 evaluators, focusing on attributes such as color, odor, texture, and flavor. The findings revealed an inverse relationship between the concentration of the EO and consumer acceptance of the sucuk, indicating that it is essential to achieve an optimal balance between product safety and consumer palatability when incorporating *T. spicata* L. EO as an alternative in sucuk production (Serhat and Yildirim 2020). In another study, Aquilani et al. (2018) performed a sensory evaluation of dry‐fermented sausages enriched with GSE, olive pomace hydroxytyrosol, and CHE, as well as olive pomace hydroxytyrosol alone. This evaluation, conducted by eight trained panelists, assessed attributes such as redness, firmness, flavor, and hardness. Notably, undesirable characteristics such as abnormal colors, off‐flavors, off‐odors, and rancidity were absent (scored as 0), while CHE samples received the highest firmness scores (scored as 9). Ultimately, although GSE and CHE products exhibited various color‐related traits, their incorporation did not compromise the overall acceptance of the sausages (Aquilani et al. 2018).

## 8 Conclusions and Future Perspectives

Meat and its derivatives stand out as valuable sources of premium proteins, essential B‐complex vitamins, and minerals. Raw meat processing, a common method for the production of various products such as sausages and ham, traditionally involves the use of cooking salts, mainly sodium nitrates and nitrites. These salts have several advantages, including color stabilization, inhibition of spoilage microorganisms such as *C. perfringens* and *C. botulinum* , and enhanced flavor and aroma of the final product. Despite their benefits, the use of healing salts raises concerns about potential health risks, particularly links to an increased risk of esophageal, stomach, and bladder cancers due to the formation of nitrosamine hormone‐like chemicals. To reduce the consumption of nitrites and nitrates, various natural alternatives such as spinach, celery, radish, lettuce, carrot, and beet have been suggested. The suggested vegetable substitutes can play different roles in fermented sausages due to their nitrate content, and it has been shown that the vegetable substitutes will increase the stability of the products due to their antimicrobial and antioxidant properties. From this category, herbal substitutes will affect the sensory profiles and general acceptance of consumers. It is suggested that future studies focused on other plant substitutes, especially those with high antibacterial properties, and exploring the physicochemical and sensory properties of the products.

## Author Contributions

All authors contributed to the study conception and design. The initial idea for the article was given by Sima Tahmouzi. The literature search and data analysis were performed by Sima Tahmouzi, Behnam Alizadeh Salmani, Soheyl Eskandari, and Masoumeh Arab. Masoumeh Arab and Sima Tahmouzi drafted and critically revised the work. All authors read and approved the final manuscript.

## Conflicts of Interest

The authors declare no conflicts of interest.

## Data Availability

Data sharing not applicable to this article as no datasets were generated or analyzed during the current study.
